# Acute posthypoxic myoclonus after cardiopulmonary resuscitation

**DOI:** 10.1186/1471-2377-12-63

**Published:** 2012-08-01

**Authors:** Aline Bouwes, Daniël van Poppelen, Johannes HTM Koelman, Michael A Kuiper, Durk F Zandstra, Henry C Weinstein, Selma C Tromp, Eveline GJ Zandbergen, Marina AJ Tijssen, Janneke Horn

**Affiliations:** 1 Department of Intensive Care, Academic Medical Center, University of Amsterdam, PO Box 22660, 1100 DD, Amsterdam, The Netherlands; 2 Department of Neurology, Sint Lucas Andreas Hospital, Amsterdam, The Netherlands; 3 Department of Neurology and Clinical Neurophysiology, Academic Medical Center, University of Amsterdam, Amsterdam, The Netherlands; 4 Department of Intensive Care, Medical Center Leeuwarden, Leeuwarden, The Netherlands; 5 Department of Intensive Care, Onze Lieve Vrouwe Gasthuis, Amsterdam, The Netherlands; 6 Alzheimer Center, Department of Neurology, VU University Medical Center, Amsterdam, The Netherlands; 7 Department of Clinical Neurophysiology, St. Antonius Hospital, Nieuwegein, The Netherlands; 8 Department of Neurology, Rijnstate Hospital, Arnhem, The Netherlands; 9 Department of Neurology, University Medical Center Groningen, Groningen, The Netherlands

**Keywords:** Coma, Anoxic, Myoclonus, Adult, Electroencephalography, Evoked potentials

## Abstract

**Background:**

Acute posthypoxic myoclonus (PHM) can occur in patients admitted after cardiopulmonary resuscitation (CPR) and is considered to have a poor prognosis. The origin can be cortical and/or subcortical and this might be an important determinant for treatment options and prognosis. The aim of the study was to investigate whether acute PHM originates from cortical or subcortical structures, using somatosensory evoked potential (SEP) and electroencephalogram (EEG).

**Methods:**

Patients with acute PHM (focal myoclonus or status myoclonus) within 72 hours after CPR were retrospectively selected from a multicenter cohort study. All patients were treated with hypothermia. Criteria for cortical origin of the myoclonus were: giant SEP potentials; or epileptic activity, status epilepticus, or generalized periodic discharges on the EEG (no back-averaging was used). Good outcome was defined as good recovery or moderate disability after 6 months.

**Results:**

Acute PHM was reported in 79/391 patients (20%). SEPs were available in 51/79 patients and in 27 of them (53%) N20 potentials were present. Giant potentials were seen in 3 patients. EEGs were available in 36/79 patients with 23/36 (64%) patients fulfilling criteria for a cortical origin. Nine patients (12%) had a good outcome. A broad variety of drugs was used for treatment.

**Conclusions:**

The results of this study show that acute PHM originates from subcortical, as well as cortical structures. Outcome of patients admitted after CPR who develop acute PHM in this cohort was better than previously reported in literature. The broad variety of drugs used for treatment shows the existing uncertainty about optimal treatment.

## Background

Many patients remain comatose after successful cardiopulmonary resuscitation (CPR) due to brain damage caused by hypoxemia. Acute posthypoxic myoclonus (PHM) occurs in about 19-37% of these patients, typically within the first 24 hours after CPR, but knowledge about this illness is limited [1,2]. Acute PHM can be divided into status myoclonus and (multi)focal myoclonus. In daily practice different terms are used for status myoclonus, e.g. generalized myoclonus, myoclonus status, or myoclonic status epilepticus [1,3,4]. Wijdicks et al. defined status myoclonus as spontaneous or sound-sensitive, repetitive, irregular brief jerks in both face and limb present during most of the first day after CPR [1]. Specific EEG criteria were not mentioned. Focal myoclonus, such as platysmal myoclonus has also been reported in patients after CPR [4]. Most studies concerning acute PHM were performed before treatment with hypothermia after CPR was implemented. Nowadays, acute PHM is often suppressed during the first day after admission by the administration of sedative drugs or neuromuscular blocking agents during hypothermia treatment. Stimulus sensitive myoclonus often interferes with nursing of these patients, is deterrent to relatives, and is difficult to treat. No evidence based guidelines for treatment exist [5-7]. Lance-Adams syndrome, chronic PHM, also occurs in patients after CPR, but usually arises in the post-intensive care unit period and it is mainly seen after hypoxia as primary cause of CPR [5,8].

Based on clinical and electrophysiological features, one can discriminate between a cortical and subcortical origin of myoclonus. Clinically, cortically generated myoclonus is mostly focal or multifocal and mainly affects parts of the body with a large cortical representation, such as hand and face. Subcortical myoclonus, especially reticular myoclonus, is generalized, predominantly affecting axial and proximal limbs. Both types of myoclonus can be stimulus sensitive [6,9-11]. Neurophysiologic investigations such as somatosensory evoked potential (SEP), electroencephalogram (EEG), electromyography (EMG), and EEG-EMG with back-averaging enable localization of the origin of myoclonus. Giant potentials on SEP recordings and epileptiform discharges or sharp/slow waves on the EEG suggest a cortical origin of the myoclonus [9,12,13]. In case of a subcortical origin of myoclonus, SEP is normal and there are no consistent abnormalities seen on the EEG [9,13].

The pathophysiology of acute PHM is poorly understood [4,5,11]. Literature suggests that acute PHM originates from subcortical and/or cortical structures [5,11]. A histological study in 15 patients with status myoclonus after CPR and 11 patients without myoclonus showed significantly more neuronal loss in all cortical laminae in patients with myoclonus, the extent of anoxic-ischemic damage in the hippocampus and cerebellum was similar in both patient groups [1].

For optimal treatment in patients with acute PHM, discrimination between cortical and subcortical origin of myoclonus would be helpful. Cortical myoclonus is primarily treated with levetiracetam or piracetam; subcortical myoclonus is usually treated with clonazepam [5-7,14].

The aim of this study was to investigate the origin of acute PHM in patients after CPR by retrospectively analyzing SEP and EEG recordings.

## Methods

Data were collected from the database of the prospective multicenter cohort study PROgnosis after PostAnoxic Coma II (PROPAC II study) [15]. In the PROPAC II study 391 adult patients admitted after CPR were included between December 2007 and August 2009. All patients were treated with hypothermia. The research protocol and consent procedures of the PROPAC II study were approved by the medical ethics committees of the Academic Medical Center (NL19644.018.07) and all collaborating hospitals: Medical Center Leeuwarden, Onze Lieve Vrouwe Gasthuis, St. Antonius Hospital, Rijnstate Hospital, Medical Center Alkmaar (METC Noord-Holland), Kennemer Gasthuis (METC Noord-Holland), Spaarne Hospital (METC Noord-Holland), Reinier de Graaf Groep (METC Zuidwest Holland), and Sint Lucas Andreas Hospital. Informed consent was obtained from a legal representative shortly after the patient’s hospital admission. When the patient regained consciousness and was able to judge the situation properly, informed consent was also obtained from the patient.

For the present study, patients reported to have focal myoclonus or status myoclonus at 24–48 and/or 48–72 hours after CPR were considered to have acute PHM. Data from those patients were retrieved from the database. Myoclonus was scored on the case record form with no further differentiation of the severity and clinical characteristics; there was no score option for multifocal myoclonus. Patients who were scored to have both focal myoclonus and status myoclonus were considered to have status myoclonus. The following variables were used: gender, age, presenting cardiac rhythm, time to return of spontaneous circulation, medical history of epilepsy, use of sedative drugs or neuromuscular blocking agents during SEP.

Neurologic outcome was assessed with the Glasgow Outcome Scale (GOS) after six months [16]. Good outcome was defined as GOS 4–5 (good recovery or moderate disability). At the time of data collection for this study, survivors were contacted by phone and asked if any form of myoclonus persisted.

Neurologic examination, including the motor score was performed 48 and 72 hours after CPR. In the PROPAC II study, cortical N20 responses of median nerve SEP were recorded with standard procedures during office hours in patients who remained comatose after regaining normal body temperature [17]. For this study, SEP recordings were retrieved and assessed by JK. The SEP was documented as *absent* (bilaterally absent cortical N20 responses after left and right median nerve stimulation, with present cervical potential), *present* (cortical N20 response present on at least one side) or *undeterminable* (technically insufficient recording). If the N20 was present, the amplitudes of the P27/N35 potentials (after right sided stimulation) were scored as < 2 μV, 2–5 μV or > 5 μV. Left-right differences were scored. Giant potentials were defined as a P27/N35 potential > 5 μV. Absent SEPs were not further analyzed.

An EEG was not a standard examination for the PROPAC II study and therefore subject to physicians’ decision. Available EEG recordings were retrieved and evaluated by JH, all EEGs were recorded during normothermia. Presence of epileptic activity, status epilepticus, generalized periodic discharges, burst suppression, isoelectric/low voltage activity (< 20 μV), and background reactivity were scored.

Neurophysiologic criteria for cortical origin of the myoclonus were giant SEP potentials or epileptic activity, status epilepticus, or generalized periodic discharges on the EEG.

Medication administered for myoclonus or epileptic seizures was reported 48 and 72 hours after CPR and categorized as: sodium valproate, clonazepam, other benzodiazepines, phenytoin, levetiracetam, and propofol. The clinical effect of the administered drugs was not evaluated.

### Statistical analysis

Patient characteristics were summarized using descriptive statistics. Variables were expressed as mean and standard deviation, or when not normally distributed, as medians and inter-quartile ranges.

## Results

In 79 of 391 (20%) patients, acute PHM (focal or status myoclonus) was reported by the physicians in the first 72 hours after CPR. The baseline characteristics and neurologic outcome are shown in Table 1. A good neurologic outcome was found in 9/77 (12%) patients after 6 months. Two patients were lost to follow-up. In these two patients, 1 month follow-up showed a good recovery in one and moderate disability in the other. There was a primary cardiac cause of CPR in 8/9 patients with a good outcome, from one patient the cause of CPR was missing. Almost all patients with a poor outcome died, one patient remained severely disabled. In the first 24–48 hours acute PHM was found in 67 patients. In 30/67 (45%) patients, acute PHM persisted at 72 hours after CPR (Table 2). Twelve additional patients developed acute PHM between 48 and 72 hours. In the majority of the patients, acute PHM was reported as focal myoclonus (47/79 patients (59%)), of whom 8 patients (17%) had a good outcome. Status myoclonus was reported in 32/79 patients (41%), 1 patient survived and made a good recovery. We were able to contact 5 of all 10 survivors by phone, one patient reported that focal myoclonus in the oral and orbital region persisted, even at 21 months after CPR. The remaining four patients had no persistent myoclonus.

**Table 1:** Baseline characteristics

		
Total number of patients, n	79	
Age, y, mean (SD)	67	(13)
Male, n (%)	58	(73)
Initial rhythm, n (%)		
VF/VT	52	(66)
Other	26	(33)
Unknown	1	(1)
Primary cause of CPR, n (%)		
Cardiac	55	(70)
Hypoxic	8	(10)
Unknown	10	(13)
Missing	6	(8)
Time from collapse to BLS, min, median, (IQR)	5	(1–10)
Time to ROSC, min, median (IQR)	15	(10–25)
Medical history of epilepsy, n (%)		
Yes	2	(3)
No	77	(97)
GOS after 6 months, n (%)		
1 Death	67	(85)
2 Vegetative state	0	(0)
3 Severe disability	1	(1)
4 Moderate disability	2	(3)
5 Good recovery	7	(9)
Unknown	2	(3)

SD = standard deviation, CPR = cardiopulmonary resuscitation, VF = ventricular fibrillation, VT = ventricular tachycardia, BLS = Basic Life Support, ROSC = return of spontaneous circulation, IQR = interquartile range, GOS = Glasgow Outcome Scale.

**Table 2:** Acute posthypoxic myoclonus

	**Focal myoclonus, n**	**(%)**	**Status myoclonus, n**	**(%)**	**Total, n**
Present 24–48 h	44	(66)	23	(34)	67
Present 48–72 h	23	(55)	19	(45)	42
Present 24–48 h and 48–72 h	20	(67)	10	(33)	30

In 64/79 (81%) acute PHM patients, electrophysiological studies were performed. SEP was recorded after rewarming in 51 (65%) patients. The results are shown in Table 3. The majority of the patients had present SEPs, only 3 patients had a giant potential on the recordings. Sixty-three percent of the patients received medication during SEP recordings. The three patients with a giant SEP potential received phenytoin, or levetiracetam in combination with sodium valproate or phenytoin.

**Table 3:** Characteristics of the performed somatosensory evoked potentials

		
Total number of performed SEPs	51	
SEP result	n	(%)
Present	27	(53)
Absent	19	(37)
Technically undeterminable	5	(10)
Left-right difference		
Yes	3	(11)
No	24	(89)
Amplitude P27/N35		
< 2 μV	21	(78)
2-5 μV	3	(11)
> 5 μV (giant potential)	3	(11)

SEP = median nerve somatosensory evoked potentials.

A total of 36 EEGs were made, results are shown in Table 4. In 23 patients (64%) EEG criteria for a cortical origin were fulfilled. Epileptiform activity and/or status epilepticus were seen in 18/36 patients (50%). Nine EEGs showed generalized periodic discharges; in 4 also epileptiform activity was seen. Only 2 patients had a burst-suppression pattern on the EEG, both patients had a reported status myoclonus. No EEGs were performed in the two patients with known medical history of epilepsy. In two of the three patients with a giant SEP potential, an EEG was performed. One EEG showed epileptic activity, the other a status epilepticus. Of the patients who underwent EEG recordings, three patients made a good recovery; none of the EEGs in these patients showed reactivity of the background, neither any of the scored EEG characteristics.

**Table 4:** Characteristics of the performed electroencephalograms

		
Total number of performed EEGs	36	
Epileptiform activity	n	(%)
Yes	12	(33)
No	24	(67)
Status epilepticus		
Yes	8	(22)
No	28	(78)
Generalized periodic discharges		
Yes	9	(25)
No	27	(75)
Burst suppression		
Yes	2	(6)
No	34	(94)
Low voltage activity/isoelectric		
Yes	2	(6)
No	34	(94)
Background reactivity		
Yes	3	(8)
No	30	(83)
Unable to assess	2	(6)
Missing	1	(3)

EEG = electroencephalogram.

The criteria for a cortical origin of the myoclonus were fulfilled in 24/64 (38%) patients in whom SEP and/or EEG was performed.

A broad variety of medication was administered (Table 5). At 48 and 72 hours, 78% (52/67) and 71% (30/42) respectively of the acute PHM patients were treated with any drug. Phenytoin, clonazepam, and propofol were used most frequently. Less than half of the patients were treated with a combination of drugs.

**Table 5:** Treatment of myoclonus

**Treatment**	**48 hours after CPR, n = 52**	**(%)**	**72 hours after CPR, n = 30**	**(%)**
Sodium valproate	14	(27)	5	(17)
Clonazepam	15	(29)	9	(30)
Other benzodiazepines	11	(21)	6	(20)
Phenytoin	15	(29)	11	(37)
Levetiracetam	10	(19)	8	(27)
Propofol	12	(23)	12	(40)
Combination	20	(38)	14	(47)
Unknown	1	(2)	0	(0)

## Discussion

The results of this study show that acute PHM originates from subcortical, as well as cortical structures; in 38% of the patients, in whom neurophysiologic investigations were performed, strong evidence towards a cortical origin of the myoclonus was found. The limited clinical information on the signs of myoclonus due to the retrospective character of the study limits further conclusions. Our data also suggest that the outcome in patients with myoclonus after CPR, irrespective of their origin, is not invariably correlated with poor outcome as previously described in literature.

To determine the origin of myoclonus, EEG and SEP recordings were used in this study. Only a very small number of patients had a giant SEP potential. A giant SEP potential is compatible with hyperexcitability of the sensorimotor cortex and proves the cortical origin of myoclonus. These giant SEPs are especially found in patients with progressive myoclonic epilepsy. However, absence of a giant SEP potential does not rule out a cortical origin of the myoclonus [13]. A reason for the low number of giant potentials of SEPs could be the flattening effect of different antiepileptic drugs, such as clonazepam, levetiracetam and sodium valproate on the amplitude of the giant SEP response [18-20]. Many patients in our study were treated with these types of drugs during SEP registration, which probably has led to an underestimation of a cortical origin.

Various EEG patterns in acute PHM patients are described in the literature; epileptic activity or sharp/slow waves on the EEG may suggest cortical origin, but does not proof it [9,12,13]. In 64% of the EEGs in our study, a cortical origin was suggested, as epileptic activity, status epilepticus, or generalized periodic discharges were seen [9]. Furthermore, bursts of generalized spikes and polyspikes or a burst suppression pattern are often found on EEG, which is thought to be consistent with severe neuronal injury [5]. Previous studies described a burst suppression pattern in 50-84% of patients with status myoclonus [1,3,4]. In our study, only 2/32 patients with status myoclonus had a burst suppression pattern. A possible explanation might be that the EEGs in our study were performed at different intervals after admission, which was previously studied by Thömke et al.. He showed that specific EEG-patterns in comatose patients with generalized myoclonus after CPR are often transient and change over time [3].

Rossetti et al. showed in a prospective cohort study of patients admitted after cardiac arrest and treated with hypothermia that 3/56 patients without EEG background reactivity survived until hospital discharge. None of these patients had a good outcome after 3–6 months [21]. On the contrary with these findings, none of the 3 patients with EEG recordings and good outcome in our study showed background reactivity.

A study limitation was that the performance of an EEG was subject to physicians’ decision. In The Netherlands, SEPs are usually performed before EEGs are requested and in case of bilaterally absent cortical N20 responses, the majority of the physicians will withdraw supportive ICU treatment, so no further investigations are performed [22]. This could have led to selection bias of patients in whom EEG was performed and it is possible that our results underestimate some of the EEG patterns in this type of patients.

In general, the outcome in our acute PHM population was better than expected based on previous publications. Several cohort studies before and after the introduction of therapeutic hypothermia after CPR reported survival rates of 0-11% for patients with acute posthypoxic myoclonus or myoclonic status epilepticus, with poor outcome for all survivors [1,3,4,23-27]. Two studies from the 1980s of Celesia et al. and Levy et al. have reported independent functioning rates similar to our study, in 1/13 (8%) and 2/21 (10%) patients respectively [28,29]. Recently, two studies in patients with myoclonus after cardiac arrest and treatment with hypothermia showed a good outcome in 3-10% [21,30]. A likely explanation for the difference in mortality between the studies is the definition used for acute PHM and status myoclonus. The original definition of status myoclonus by Wijdicks et al. included revelation within 24 hours. Nowadays, acute PHM might be suppressed during hypothermia treatment due to administration of sedative drugs. Another possible explanation might be the limited number of patients with myoclonus in the studies.

Acute PHM is difficult to treat and knowledge about the origin in the brain could probably lead to better treatment and identification of patients with a more favourable outcome. In our study, various drugs were used for the treatment of acute PHM. This reflects the uncertainty about the best treatment for this syndrome [5,14]. In general, cortical myoclonus is primarily treated with levetiracetam or piracetam; subcortical myoclonus is usually treated with clonazepam [5-7,14]. Treatment with phenytoin, sodium valproate, phenobarbital or various benzodiazepines is often ineffective [1,3,25]. Propofol causes enhancement of gamma-amino butyric acid and has been successfully used as treatment of acute PHM [25,31].

A study limitation is the retrospective method used to select patients from the PROPAC II study, which was designed to establish the validity of diagnostic methods to predict poor outcome in patients treated with mild hypothermia after CPR. The fact that not in all patients neurophysiologic tests were performed is also a limitation and might limit robust conclusions from this study. A study primarily investigating acute PHM should ideally contain video recording of the myoclonus in each patient, evaluation by blinded assessors and strict evaluation of the effect of standardized medication used. Furthermore, it should combine EEG and SEP recordings with EEG-EMG with back-averaging, as this last method can prove a cortical origin [13]. Future research with the above mentioned standardized observation techniques, more neurophysiologic tests, and randomized treatment groups are necessary to evaluate the effect of different treatment strategies.

## Conclusions

Despite the limitations mentioned above we think that acute PHM originates from subcortical, as well as cortical structures and that the outcome of patients admitted after CPR who develop acute PHM might be better than previously reported. The broad variety of drugs used for treatment shows the existing uncertainty about optimal treatment.

## Competing interests

Dr. Horn received a research grant from the Dutch Heart Foundation (2007B039). Dr. Tijssen received research grants and travel/accommodations/meeting expenses as money for the institution in the previous 12 months. Dr. Bouwes, Dr. van Poppelen, Dr. Koelman, Dr. Kuiper, Dr. Zandstra, Dr. Weinstein, Dr. Tromp and Dr. Zandbergen report no disclosures.

## Authors’ contributions

AB, DP, JK, MT, JH have made substantial contributions to conception and design, and analysis and interpretation of data. AB, DP, JK, MK, DZ, HW, ST, EZ, MT, JH have made substantial contributions to acquisition of data and have been involved in drafting the manuscript or revising it for important intellectual content. All authors have given final approval of the version to be published.

## Pre-publication history

The pre-publication history for this paper can be accessed here:

http://www.biomedcentral.com/1471-2377/12/63/prepub
